# Mining literature and pathway data to explore the relations of ketamine with neurotransmitters and gut microbiota using a knowledge-graph

**DOI:** 10.1093/bioinformatics/btad771

**Published:** 2023-12-26

**Authors:** Ting Liu, K Anton Feenstra, Zhisheng Huang, Jaap Heringa

**Affiliations:** Integrative Bioinformatics, Vrije Universiteit Amsterdam, Amsterdam 1081 HV, The Netherlands; Learning & Reasoning Group, Vrije Universiteit Amsterdam, Amsterdam 1081 HV, The Netherlands; Integrative Bioinformatics, Vrije Universiteit Amsterdam, Amsterdam 1081 HV, The Netherlands; Learning & Reasoning Group, Vrije Universiteit Amsterdam, Amsterdam 1081 HV, The Netherlands; Integrative Bioinformatics, Vrije Universiteit Amsterdam, Amsterdam 1081 HV, The Netherlands

## Abstract

**Motivation:**

Up-to-date pathway knowledge is usually presented in scientific publications for human reading, making it difficult to utilize these resources for semantic integration and computational analysis of biological pathways. We here present an approach to mining knowledge graphs by combining manual curation with automated named entity recognition and automated relation extraction. This approach allows us to study pathway-related questions in detail, which we here show using the ketamine pathway, aiming to help improve understanding of the role of gut microbiota in the antidepressant effects of ketamine.

**Results:**

The thus devised ketamine pathway ‘KetPath’ knowledge graph comprises five parts: (i) manually curated pathway facts from images; (ii) recognized named entities in biomedical texts; (iii) identified relations between named entities; (iv) our previously constructed microbiota and pre-/probiotics knowledge bases; and (v) multiple community-accepted public databases. We first assessed the performance of automated extraction of relations between named entities using the specially designed state-of-the-art tool BioKetBERT. The query results show that we can retrieve drug actions, pathway relations, co-occurring entities, and their relations. These results uncover several biological findings, such as various gut microbes leading to increased expression of BDNF, which may contribute to the sustained antidepressant effects of ketamine. We envision that the methods and findings from this research will aid researchers who wish to integrate and query data and knowledge from multiple biomedical databases and literature simultaneously.

**Availability and implementation:**

Data and query protocols are available in the *KetPath* repository at https://dx.doi.org/10.5281/zenodo.8398941 and https://github.com/tingcosmos/KetPath.

## Introduction

Depression is a common mental health problem that affects an estimated 5% of the global population and has a lifetime prevalence of up to 20% of individuals (Gutiérrez-Rojas *et al.* 2020). As an antidepressant, ketamine triggers an increased release of various neurotransmitters, which promote the growth of certain gut bacteria and, conversely, are synthesized and/or consumed by gut microbiota (see Supplementary Fig. S1A). Moreover, it is well known that neurotransmitters and gut microbiota are associated with depression (Cheung *et al.* 2019). Taken together, these facts suggest that gut microbiota may be involved in the antidepressant effects of ketamine by modulating neurotransmitters. Although some studies have found a link between gut microbiota and ketamine’s antidepressant action (Wilkowska *et al.* 2021), current knowledge remains too limited for understanding the role of specific gut microbiota species in the ketamine pathway. At present, only a few publications address the co-action of ketamine and gut microbiota in depression (see Supplementary Fig. S1B). To further our knowledge, uncovering possible pathways to explore the role of neurotransmitters and gut microbiota in ketamine’s actions is crucial.

Biomedical pathways are generally provided in the form of (i) computer actionable structured datasets in public databases or (ii) unstructured human-readable static images and free texts in scientific articles. Existing databases are useful resources as they have collected diverse data on pathways, drugs, genes, and microbes, but their information on specific pathways lags behind new insights in the latest articles. This knowledge gap results in the need to aggregate information not only from available databases but also from pathway images in the literature, where the latter requires great effort in manual curation.

Aggregating existing databases brings together disparate biomedical data and establishes links between them, thereby enriching the underlying knowledge and facilitating the formulation of data-searching strategies. As summarized in Supplementary Fig. S1C, this study uses several public databases that hold community-accepted and relevant medical terms. For example, UMLS and SNOMED CT are used as thesauri for identifying named entities from plain text (Stearns *et al.* 2001, Bodenreider 2004), while the KEGG database is adopted to deliver data profiles of pathways, genomes, and compounds (Kanehisa *et al.* 2014). These databases are tied together in a knowledge graph.

Knowledge graphs enable the representation of diverse information as a collection of nodes (entities) and edges (relations), resulting in flexible structures that adapt to complex data and enable the effective use of network analysis techniques to identify hidden patterns and knowledge (Santos *et al.* 2022). They hold the advantage of integrating information from disparate research resources, which may lead to new insights. They have been widely adopted and have shown promising applications in biomedical areas, such as disease classification (Fang *et al.* 2019), drug discovery (Sang *et al.* 2018), adverse drug reactions (Bean *et al.* 2017), target prediction (Mohamed *et al.* 2020), and combination therapies (Du and Li 2020). In our previous studies, we explored the use of knowledge graphs to collect and explore existing knowledge on gut microbiota. We constructed a microbiota knowledge graph (MiKG) to represent the relationship between gut microbiota and neurotransmitters by manually curating relations (Liu *et al.* 2020) and a vastly larger pre-/probiotics knowledge graph (PPKG) to express the relationship between pre-/probiotics and human diseases by combining manual relation curation with named entity recognition (Liu *et al.* 2022). In the latter work, we used XMedlan, a Xerox Concept Identifier (CI-er) tool for named entity recognition and semantic annotation of biomedical text (Hu *et al.* 2015). It tracks the co-occurrence of entities by answering queries such as ‘retrieve all sentences where depression and ketamine co-occur’, irrespective of a possible relation between them.

This work takes the manual curation and named entity recognition methods from previous works and further extends these with natural language processing techniques for automatic relation extraction. This approach would enable us to complement relations from the public databases with novel relations from the latest literature. This study employs BioBERT (Lee *et al.* 2020), a biomedical language representation model pre-trained on biomedical domain corpora (i.e., PubMed abstracts and PMC full-text articles) that achieves state-of-the-art performance in relation extraction tasks, to derive pathway relations from biomedical text.

Using the combination of manual relation curation, named entity recognition, and relation extraction, we construct the ketamine pathway ‘KetPath’ dataset comprising three subsets: (i) KetFact, containing manually curated pathway facts from images; (ii) KetCept, consisting of concepts (named entities) recognized using CI-er; and (iii) KetRela, covering relations between named entities identified by BioBERT. This dataset is integrated into the KetPath knowledge graph along with the MiKG, PPKG, and multiple public databases. The resulting knowledge graph is stored in the GraphDB platform for information retrieval.

We present four query cases based on biological questions to retrieve meaningful results and thereby validate the usefulness of the KetPath knowledge graph. We believe that the resources and findings established in this work will aid both (i) the biomedical researchers who wish to investigate ketamine’s antidepressant actions and (ii) the knowledge graph researchers who intend to facilitate the application of knowledge graphs in the biomedical domain.

## Methods

**Figure 1.**
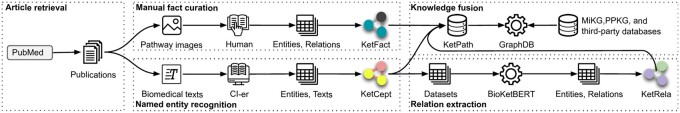
Approach for constructing the KetPath knowledge graph. The KetPath knowledge base is built by combining manual curation (KetFact), automatic named entity recognition (KetCept) with CI-er (Hu *et al.* 2015), and automatic relation extraction (KetRela) with BioKetBERT, which is a fine-tuned BioBERT model (Lee *et al.* 2020). It is stored in GraphDB, a graph analytics platform that supports data access using the SPARQL query language, along with the MiKG and PPKG knowledge bases and multiple public databases.

We construct the KetPath knowledge graph by consolidating data from scientific articles and public databases. Our approach consists of five main steps as illustrated in Fig. 1. In the following sections, we will describe each step in detail.

### Article retrieval

We retrieved 2773 articles from PubMed on 1 September 2022, using ketamine and depression as keywords in the following query: https://eutils.ncbi.nlm.nih.gov/entrez/eutils/esearch.fcgi?db=pubmed&term=“Ketamine+Depression”&RetMax=10000. The websites of the retrieved articles were stored locally in HTML format, expressly limited to the title and abstract. For instance, the web URL “https://pubmed.ncbi.nlm.nih.gov/21907221/” corresponds to the article with PMID 21907221. Furthermore, the pathway images and their captions or related texts describing the ketamine pathway in the relevant articles were stored in JPG or TXT formats for further manual pathway fact curation and automatic named entity recognition, respectively.

### Manual fact curation from pathway images

Ketamine pathways are often spread across publications as static images and free texts, as shown in Supplementary Fig. S2A. We extract pathway facts, i.e., entities and relations, from publication figures. They generally appear as a graph containing a number of entities connected by relational expressions and reaction expressions specifying substrates and products, which we normalize into semantic triples in the form of subject–predicate–object expression as T=(S,P,O).

**Figure 2.**
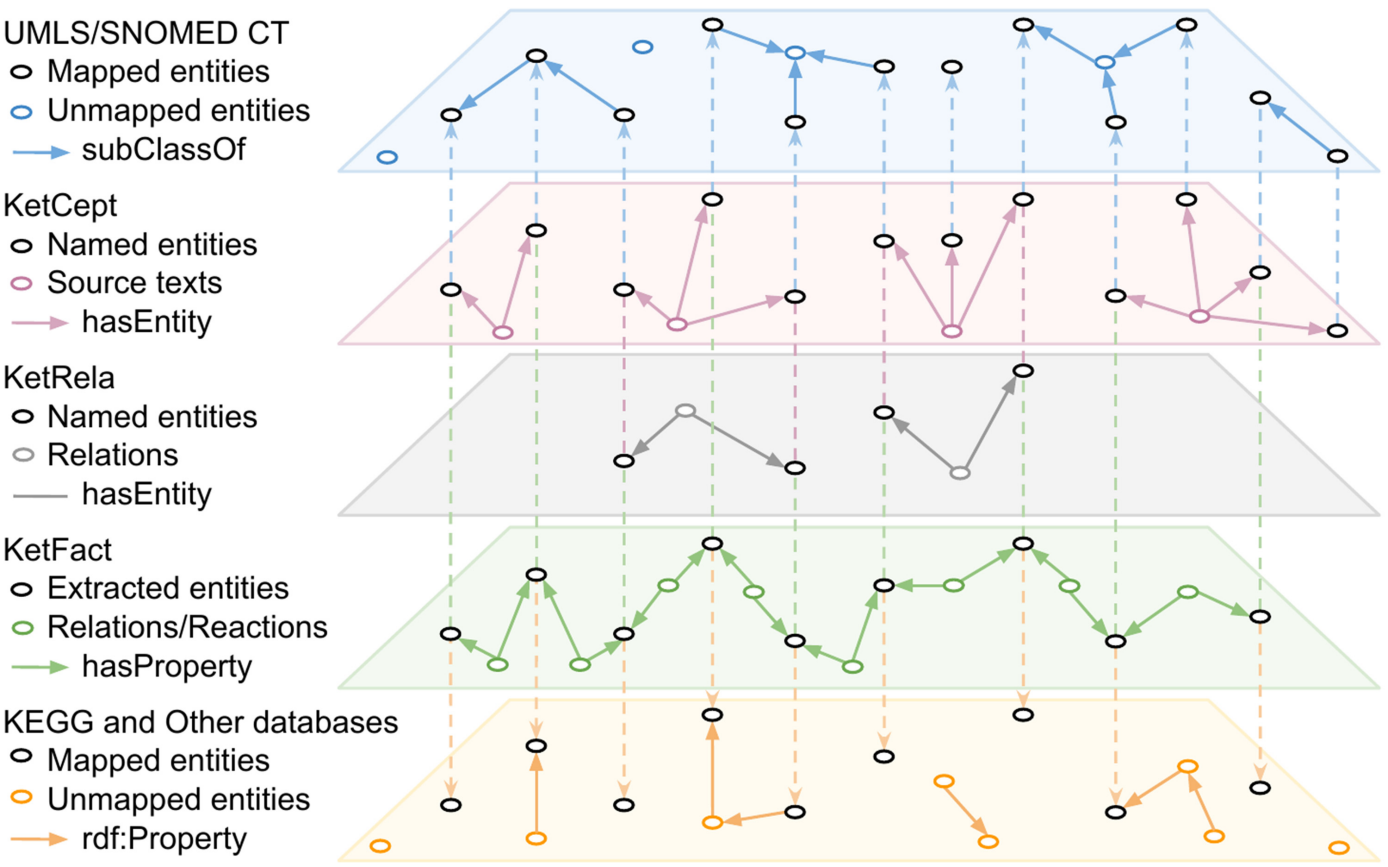
Entity mapping across datasets. The five layers correspond to the datasets used; from top to bottom: UMLS/SNOMED CT, KetCept, KetRela, KetFact, KEGG, and other databases. Entities are nodes, connected by relations shown as edges. Entity mappings between datasets are depicted as dashed lines. Not all entities are present in all data layers, and for some connections, multiple aspects are described in different layers.

Entities and relations are manually extracted from the image, in which relations are divided into activation (arrow-headed lines) and inhibition (bar-headed lines), as in the example shown in Supplementary Fig. S2B. Entities are mapped to existing entries in the KEGG database by matching their Uniform Resource Identifiers (URIs); matches were verified by checking their description and pathway connections. This is shown in the bottom two layers of Fig. 2, where KetFact entities (nodes in the second bottom layer) and relations (edges) map (dotted lines) onto KEGG entities (nodes in the bottom layer) and relations (edges). A relation expression serves as a subject entity that connects two object entities, thereby depicting the relationship between them, as shown in Supplementary Figs S2B and S3. The same is true of a reaction expression which also acts as an entity and is connected to entities that represent substrates, products, and other reaction annotations, as the example shown in Supplementary Fig. S3. These manually extracted pathway facts constitute KetFact (see Fig. 1).

### Named entity recognition from biomedical texts

CI-er was developed for term identification based on sequential strategies combining typographical, morphological, orthographical, and misspelling normalization, mainly implemented with compositions of finite-state transducers. It performs well in recognizing entities from biomedical text with evaluation metrics precision, recall, and F1 score of 92% (Hu *et al.* 2015). We used this tool to annotate the titles and abstracts of the retrieved articles, as well as the captions and any relevant text pertaining to the pathway images. Named entities were annotated by CI-er with an entity URI by matching words to the associated entity labels in UMLS and SNOMED CT. This is shown in Supplementary Fig. S2C and the top two layers of Fig. 2, where KetCept entities (nodes in the second top layer) map (dotted lines) onto UMLS/SNOMED entities (nodes in the top layer). Overall, a scientific paper P is represented by multiple annotations A, as $P=[A_{1},A_{2},\ldots ,A_{n}]$, and each annotation $A_{i}$ consists of the source sentence $S_{i}$ with extracted entities E, as $A_{i}$ =$\left[S_{i},E_{i,1},E_{i,2},\ldots ,E_{i,n}\right]$, as depicted in Supplementary Fig. S2C. These annotations constitute the KetCept dataset (see Fig. 1).

## Relation extraction

### Relation extraction with BioBERT and BioKetBERT

Relation extraction is the task of identifying relations between named entities in biomedical texts. We used BioBERT, a state-of-the-art language model pre-trained on biomedical domain corpora (Lee *et al.* 2020), to perform relation extraction tasks on a dataset containing 2143 sentences retrieved from the KetCept dataset through semantic queries. We prepared these sentences as described by Lee *et al.* (2020). The target-named entities were tagged using pre-defined tags as @entity$. For instance, the drug ‘ketamine’ in sentences is replaced by @ketamine$ and ‘serotonin’ by @serotonin$. Entity tagging was performed for each relation, meaning each sentence contains only one relation, i.e., only one pair of entities. Finally, a label indicating whether the two entities in a sentence are related has been added: 1 for a relation, 0 otherwise. We dubbed the resulting model BioKetBERT, and the relations extracted with it constitute the KetRela dataset (see Fig. 1). Figure 2 shows the mapping of entities (nodes) in KetRela to those in KetCept.

### Validating BioKetBERT

Since we optimized BioBERT for building our ketamine knowledge graph, yielding the BioKetBERT model, it is essential to validate its performance. In this study, the performance of relation extraction tasks was evaluated using five-fold cross-validation, where the dataset containing 2143 sentences was randomly partitioned into five stratified subsets of equal size: for each round, one block (428 sentences) was used for training and the remaining (1715 sentences) four were for testing. We evaluated these tasks by reporting recall, precision, specificity, and F1 score (see section S2 for details). The evaluation results of all tasks were averaged over five runs, along with their standard deviations. We also compared the evaluation results of BioKetBERT with CI-er as a baseline. Since CI-er can recognize named entities but cannot perform relation extraction, we have added two naive approaches as a baseline to compare with (i) CI-er-1 assumes no relations between named entities and (ii) CI-er-2 assumes all named entities within a text are related.

## Knowledge fusion

### Knowledge graph construction

We built a knowledge graph database called KetPath in GraphDB to integrate and connect pathway datasets (see Fig. 1). The data in GraphDB can be accessed and reasoned over using the SPARQL query language (Güting 1994), which is a popular query protocol for retrieving and manipulating structured data in RDF format (Lan *et al.* 2022).

### Data sources and contents

The KetPath knowledge graph contains datasets derived from two types of resources: scientific articles and public databases. Supplementary Fig. S4 illustrates the connections between these datasets. The three datasets derived from publications are (i) KetPath, representing pathway knowledge of ketamine; (ii) MiKG, regarding relations between gut microbiota and neurotransmitters (Liu et al. 2020); and (iii) PPKG, featuring relations between pre-/probiotics and human diseases (Liu *et al.* 2022). We integrated the knowledge graph with seven public databases (Supplementary Fig. S1C): (i) WikiPathways for human pathways of chemical signalling and drug metabolism; (ii) KEGG for 70 pathway maps of *homo sapiens* and compound profiles; (iii) DrugBank for drug actions (Wishart *et al.* 2018); (iv) Gene Ontology for gene and gene attributes (Gene Ontology Consortium 2021); (v) UniProt for human protein sequences and annotations (UniProt Consortium et al. 2018); (vi) UMLS (Bodenreider 2004); and (vii) SNOMED CT thesaurus (Stearns *et al.* 2001) for recognizing named entities. These datasets are divided into terminological components (*TBox*), which describe the domain of interest by defining classes and properties as schemas, and assertional components (*ABox*), which are *TBox*-compliant facts related to their conceptual ontologies. An example is shown in Supplementary Fig. S4, where the statement ‘*Gene makes proteins*’ is a *TBox*, and ‘*Ketamine is a drug*’ is an *ABox*.

### Normalizing KEGG pathways

Some public databases provide endpoints for online queries, such as WikiPathways and DrugBank, while others do not and their data are not in RDF format, such as KEGG. KEGG provides pathway maps in KGML format, which were converted to RDF format using a custom-created XSLT stylesheet, conforming to the example shown in Supplementary Fig. S3. We designed an RDF schema to capture information from KGML, mainly including entries, relations, reactions, substrates, and products. To illustrate, we defined a set of properties for representing a *reaction* with properties *hasSubstrate*, *hasProduct*, *ReactionType*, and a linkage to KEGG, as can be seen in Supplementary Fig. S3. Based on the structure of KGML, we created an XSLT stylesheet that extracts information from KGML and maps it to the RDF schema, generating RDF pathway data.

### Using the knowledge between datasets

Entity alignment is an essential process that aims to unify the representation of entities as diverse terms that exist in various databases referring to the same entity. To achieve this, we mapped entities between datasets by matching their URIs with the *owl: sameAs* statement. For example, the mapping of entity *serotonin* in the KetFact knowledge base to the URIs of serotonin in the KEGG and UMLS databases is represented as ‘*ketfact: Serotonin owl: sameAs kegg: C00780, umls: C0036751*’. Figure 2 shows the mapping relation of entities between different datasets.

## Results

### Data collection and knowledge graph construction

KetPath aggregates ten datasets (see Supplementary Fig. S1C) derived from two types of sources: (i) scientific publications, offering up-to-date but unstructured knowledge of the ketamine pathway, and (ii) existing databases, providing well-structured information on the underlying biomedical data.

The data used to construct KetPath comprise three subsets: (i) KetFact, containing 36 entities and 50 relations for ketamine pathway facts that were manually curated from images; (ii) KetCept, including 218 710 named entities from 25 280 sentences that were identified using CI-er; and (iii) KetRela, comprising 2143 relations extracted using BioKetBERT. Supplementary Table S1 summarizes data statistics of these three as well as the other existing datasets and databases included in the KetPath knowledge graph. We included our previously constructed MiKG (Liu *et al.* 2020), which holds a total of 2175 relations between gut microbes and neurotransmitters, and PPKG (Liu *et al.* 2022), which consists of *ppstatement* containing 446 manually annotated statements of pre-/probiotics treatment details and *ppconcept* comprising 2 307 359 named entities recognized from 291 492 sentences. Furthermore, we included seven structured third-party databases on specialist subjects as detailed in Methods.

### Improving and evaluating relation extraction

In order to automatically obtain the relations for the KetRela dataset, we fine-tuned the BioKetBERT model and compared its performance to the general BioBERT relation extraction model provided by Lee *et al.* BioKetBERT was fine-tuned based on the data from the first query case, as explained in the next section. We evaluated the performance of the two models and reported their F1, recall, precision, and specificity scores, as shown in Table 1. Finally, the prediction and evaluation results of all tasks were averaged over the five runs.

**Table 1.** Estimating relation extraction performance of different models on test sets.

Model	TP	FP	TN	FN	F1 (%)	Recall (%)	Precision (%)	Specificity (%)
CI-er-1	0	0	876	1267	0	0	0	**100**
CI-er-2	1267	876	0	0	74	**100**	59	0
BioBERT-5×CV	963±73	541±113	160±120	51±46	76±2	95±5	64±4	23±17
BioKetBERT-5×CV	902±22	228±94	473±56	112±51	84±2	89±5	80±6	68±11
BioBERT	1266	870	6	1	74	**100**	59	1
BioKetBERT^a^	1186	135	741	81	**92**	94	**90**	85

CI-er (Hu *et al.* 2015) does not extract relations from a text, here CI-er-1 assumes no relations between named entities, and CI-er-2 assumes all named entities within a text are related. The highest score for each metric is bolded. 5×CV means five-fold cross-validation, with average values and standard deviations reported.

TP, true positives; FP, false positives; TN, true negatives; FN, false negatives.

a The final model was fine-tuned on the complete dataset; reported metrics are thus on the whole set.

A total of 2143 sentences containing ketamine and at least one neurotransmitter retrieved from the KetCept dataset were used for fine-tuning and testing. As a result, CI-er-1 yields 876 TNs and 1267 FNs, whereas CI-er-2 leads to 1267 TPs and 876 FPs (see also Table 1). We see a high variation in specificity for both the BioBERT and BioKetBERT models, which is caused by the relatively small number of TN and a high variation of FP (see Table 1). A higher specificity score signifies an improved identification of true negatives, which is crucial for avoiding false positives and thus ensuring accurate identification of the precise relationships between entities. Notably, the F1 score of BioBERT at 76% is barely higher than that of CI-er-2 with F1 at 74%; similarly for the precision: 64% over 59%. In particular, we see the over-prediction of BioBERT from the high FP and low precision in Table 1. Overall, the general BioBERT model does not perform much better than the naive CI-er-2 approach for this application. Overall, BioKetBERT is much better than both approaches, achieving an F1 of 84% and a precision of 80%. In Supplementary Table S2, we present a breakdown of individual ketamine–neurotransmitter relations predicted by BioBERT and BioKetBERT, which shows that performance is fairly unbiased across these neurotransmitters. Supplementary Table S3 shows further statistics of this query result per neurotransmitter, in which a total of 1186 sentences were labelled by both BioBERT and BioKetBERT as containing a ketamine–neurotransmitter relation consistently with the manual labels. Overall, BioKetBERT performs well in identifying relations between ketamine and neurotransmitters. To make the KetRela dataset more biologically comprehensive, all relation data manually labelled by experts or predicted by either model are included in the KetRela dataset, thus allowing users to retrieve all combinations of manually annotated relations and those predicted by BioBERT and BioKetBERT.

### Knowledge graph validation and information retrieval

We present four query cases, each with a corresponding biological question, to validate the usefulness of the integrated KetPath knowledge graph and retrieve meaningful results for biological questions.

### Which neurotransmitters does ketamine affect?

Query case 1 answers the question: *which neurotransmitters does ketamine affect?* The corresponding query code to retrieve these relations is shown in Supplementary Listing S1. The query code retrieved 2143 sentences containing the named entities ketamine and at least one neurotransmitter from the KetCept dataset, as well as 2143 predicted relations from the KetRela dataset. These sentences and relations are derived from 1041 related articles, as shown in Supplementary Table S3, which points out the associations between ketamine and neurotransmitters. The highest number of relations, where manual predictions and those by BioBERT and BioKetBERT are consistent, is ketamine–glutamate with 504, and the two lowest are acetylcholine and histamine with 14 and 12, respectively.

### How does ketamine affect neurotransmitters?

The results of query case 1 summarize what is known about the relationship between ketamine and neurotransmitters. To explore this relationship further, query case 2 aims to answer the question ‘how does ketamine affect neurotransmitters?’. The corresponding query code is shown in Supplementary Listing S2. The first step retrieves drug actions with specific entities involved. The second step adds the pathway graphs from public databases included in the KetPath knowledge graph.

**Figure 3.**
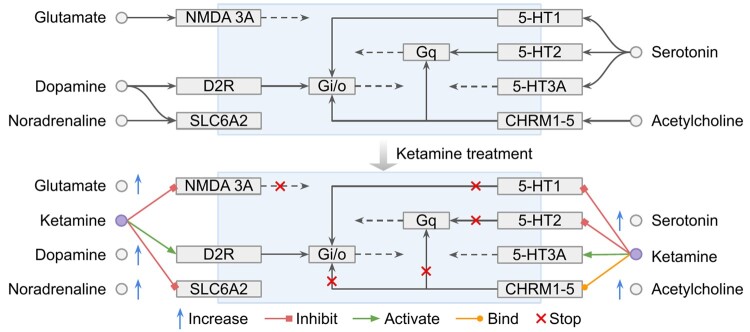
Summarizing the result of query case 2 as a biological action, demonstrating the main effects of ketamine on neurotransmitter pathways. Pathways are summarized as their neurotransmitter and main interactions. The top panel shows the pathways derived from the KEGG and WikiPathways databases, while the bottom panel illustrates the effect of ketamine on these pathways as retrieved from KetFact.

The query code retrieved seven pharmacological actions of ketamine acting in multiple capacities on specific receptors or transporters of five neurotransmitters from the DrugBank database, as shown in Supplementary Table S4. This code is able to return the elements—entries, relations, and reactions—in pathway graphs. The entire query results comprise 273 relations along with 471 entities from the KEGG and WikiPathways, which is too large to show in detail, and we present the first 20 returned results in Supplementary Table S5. Figure 3 summarizes the main effects, which give us an insight into how ketamine affects the five neurotransmitters and their corresponding pathways. In short, ketamine acts on specific receptors and transporters of neurotransmitters, thus increasing free glutamate, dopamine, serotonin, noradrenaline, and acetylcholine.

What are the pathway relations for ketamine in the literature?

Query case 3 aims to answer the question “What are the pathway relations for ketamine in the literature?” by retrieving the pathway relations from the KetFact dataset, which are manually collated from published figures in the literature. The associated code is reproduced in Supplementary Listing S3.

**Figure 4.**
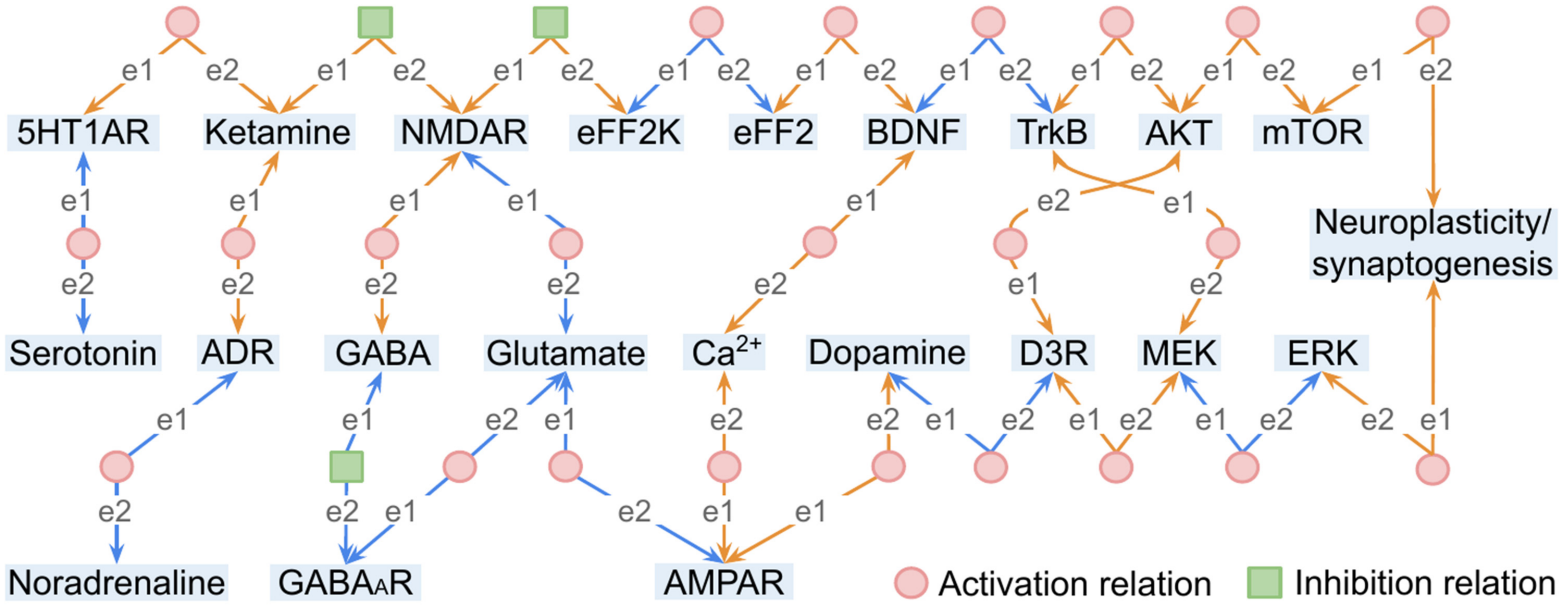
The retrieved relations in the ketamine pathway in literature by query case 3. The corresponding query code is shown in Supplementary Listing S3. Green squares indicate inhibition and red circles indicate activation. All relations are from Entity 1 (e1) to Entity 2 (e2). Blue lines indicate relations in the KEGG databases, while orange lines are only in KetFact.

This query returned 26 relations linking 22 entities, as shown in Fig. 4. Within KetPath, entities are mapped between KetFact and KEGG, enabling us to perform analysis across database-derived and literature-derived pathway graphs, as retrieved in use cases 2 and 3, respectively. Of the 26 relations retrieved here that are based on literature, ten (blue lines) already exist in the public databases, while the other 16 (orange lines) can only be found in the literature, as shown in Fig. 4. Of the latter, five relations also correspond to indirect relations through the pathway graphs derived from KEGG. Thus, these query results indicate that the KetPath KG is more biologically comprehensive than only the KetRela. For example, we can now conclude critical novel findings such as that several neurotransmitters, notably glutamate, play a crucial role in the ketamine pathway or that ketamine indirectly affects the BDNF, which is essential for activating downstream signalling.

### How are gut microbes, neurotransmitters, BDNF, and ketamine related?

**Figure 5.**
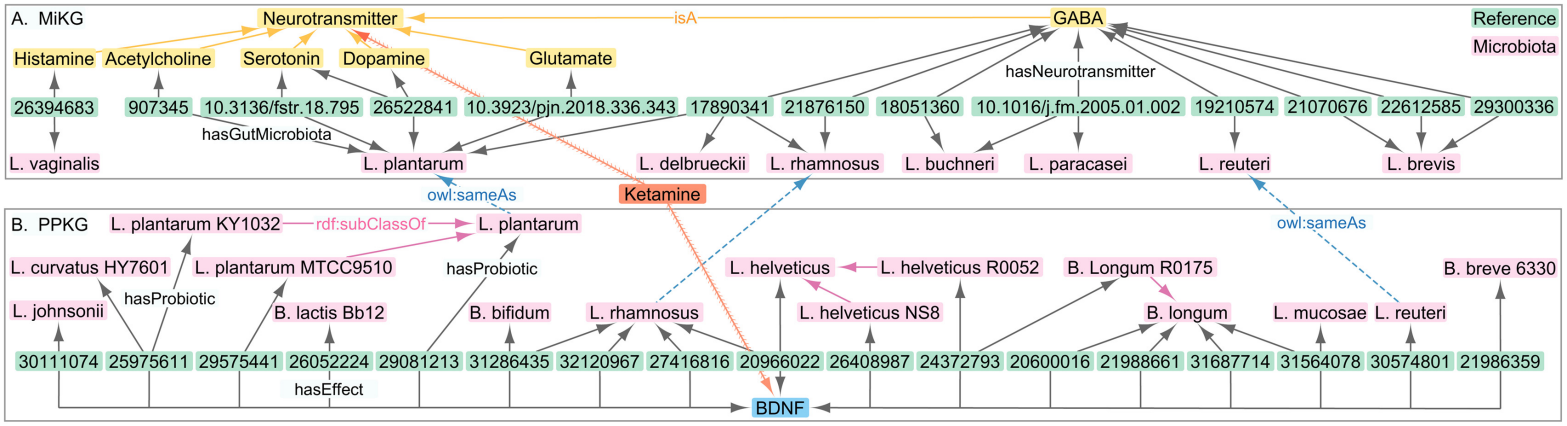
The returned relations between ketamine and gut microbiota by query case 4. The query code is presented in Supplementary Listing S4. (A) Retrieved the neurotransmitter-mediated relations between ketamine and gut microbiota from MiKG (Liu *et al.* 2020). (B) Retrieved the BDNF-mediated relations between ketamine and gut microbiota from PPKG (Liu *et al.* 2022). References are indicated by their PMIDs or DOIs. *Bifidobacterium* is abbreviated as *B.* and *Lactobacillus* is shortened to *L*.

The preceding query cases have shown that neurotransmitters and BDNF play a vital role in the ketamine pathway, and long-term clinical use of ketamine strikingly amplifies the *Lactobacillus*, *Turicibacter*, and *Sarcina* genera (Getachew *et al.* 2018). Query case 4 hence retrieves the neurotransmitter- and BDNF-mediated relations between ketamine and gut microbiota, of which the query code is presented in Supplementary Listing S4. This query obtained a total of 30 articles. Of these, 13 derived from the MiKG knowledge base indicated that gut microbes increase neurotransmitter contents. The remaining 17 articles originated from the PPKG knowledge base and indicated that gut microbes affect BDNF levels, as shown in Fig. 5. The figure shows that various *Lactobacillus* strains lead to increased neurotransmitter levels, especially GABA, followed by serotonin, glutamate, dopamine, acetylcholine, and histamine. Moreover, various gut bacteria used alone or in combination promote BDNF expression. Most of these bacteria belong to the genus *Lactobacillus*, while the rest belong to the genus *Bifidobacterium*. Overall, this query case provides evidence for both neurotransmitters and BDNF-mediated relations between ketamine and gut microbiota.

### Summarizing biological pathways from query results

**Figure 6.**
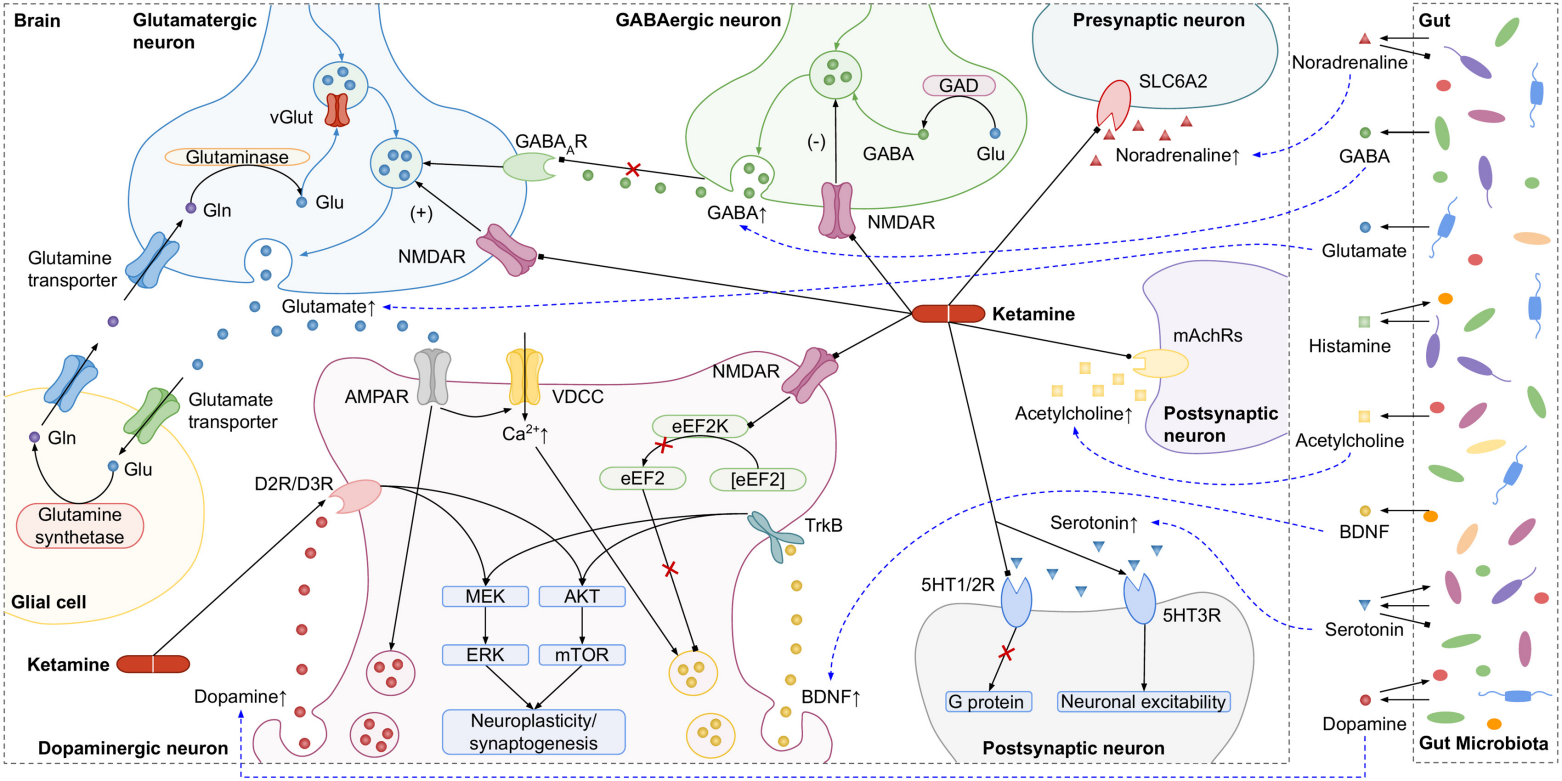
Summarizing the ketamine pathway and the role of gut microbiota in it from the results of all query cases. The relations of gut microbes with BDNF and neurotransmitters shown on the right part were retrieved from MiKG (Liu *et al.* 2020) and PPKG (Liu *et al.* 2022), as shown in detail in Fig. 5. Black arrow-headed lines indicate activation, black square-headed lines mean inhibition and black round-headed lines represent binding. Red crosses indicate blocking. Blue dashed arrows connect BDNF and neurotransmitters produced by gut microbiota to those in the ketamine pathway. Molecular entities in this figure are defined in Supplementary Table S6 with their abbreviation, full name, and KEGG entry.

We summarize the biological findings gleaned from the four query cases below (also see Fig. 6 and molecular entity abbreviations in Supplementary Table S6):

- Ketamine increases synaptic glutamate release by blocking NMDARs either on GABAergic or glutamatergic synapses. Blocking NMDARs on GABAergic neurons reduces their inhibitory input on glutamatergic neurons, and blocking NMDARs on glutamatergic neurons promotes their excitatory synaptic drive.
- The burst release of glutamate activates AMPARs on dopaminergic neurons, which leads to the activation of VDCC, causing two effects. First, increased dopamine release promotes D2R/D3R signalling, which can also be activated by ketamine. Secondly, enhanced BDNF/TrkB signalling results in increased extracellular free BDNF.
- Blockage of NMDARs on dopaminergic neurons by ketamine leads to the inactivation of eEF2 kinase. Such inactivation prevents the phosphorylation of eEF2, thereby reducing its content. The reduction of eEF2 prevents its inhibition of BDNF/TrkB signalling.Increased D2R/D3R signalling and BDNF/TrkB signalling activate downstream MEK/ERK and AKT/mTOR pathways, which drive the molecular machinery required for neuroplasticity and synaptogenesis.
- Inhibition of the noradrenaline transporter by ketamine prevents its uptake of noradrenaline at the gap junctions, leading to an increase in free noradrenaline. Both extracellular serotonin and acetylcholine are increased caused by the action of ketamine on their receptors.
- All these neurotransmitters and BDNF are essential in the ketamine pathway and can be produced or promoted by gut microbes. In turn, some can stimulate or inhibit the growth of certain gut bacteria.

As shown in Fig. 6, the connection between the brain and the gut related to ketamine’s actions is established through BDNF and neurotransmitters, which are critical for the sustained antidepressant effects of ketamine.

### Reusing the repository and queries

The datasets established in this work are available at the github. KetPath respiratory on GitHub. We provide a *README* file to illustrate integrating multiple datasets into a knowledge graph. The first step is to create a repository named KetPath on the GraphDB platform. Afterwards, users need to download the datasets from GitHub and upload them to the KetPath repository as named graphs with unique URLs. For example, we set the KetFact dataset as a named graph with URL http://wasp.cs.vu.nl/ketfact. Users are allowed to customize the URL for each dataset. Other custom datasets can be integrated into the knowledge graph similarly. Data in GraphDB can be accessed and reasoned about using the SPARQL protocol. We provide several query protocols in the Supporting Information, encoded in the SPARQL language. Users can easily create their queries using the provided protocols as templates and changing parameters or extending the query as needed. These query protocols are composed of basic query clauses and each contains less than 30 variables, taking less than half a minute to retrieve data from the knowledge graph.

## Discussion

The KetPath knowledge graph integrates knowledge of ketamine pathways from public databases and scientific publications. It contains knowledge at three different levels of description: (i) manually extracted ketamine pathway facts from images in KetFact, (ii) automatically identified named entities from unstructured texts in KetCept, (iii) automatically extracted relations between named entities in KetRela, and (iv) multiple community-accepted public databases. It thus ensures the accuracy and completeness of the query results and provides added value.

We validate the usefulness of KetPath with four query cases based on biological questions, showing that each can produce multiple different meaningful results, such as:

- query descriptions of entities across different databases (Fig. 3);
- fetch core entities, relations, and reactions of pathways (Fig. 4);
- obtain article sentences covering entities of interest (Fig. 5);
- retrieve pharmacological targets and actions of drugs (Supplementary Table S4);
- gather the relations between ketamine, neurotransmitters, and gut microbiota (Fig. 5).

This last result also shows how various gut bacterial strains used alone or in combination can increase neurotransmitter levels and/or promote *bdnf* expression (Fig. 5). Interestingly, the two most involved genera, i.e., *Lactobacillus* and *Bifidobacterium*, are known to be associated with depression (Yong *et al.* 2020). Conversely, the reversal of depressive symptoms by ketamine usually accompanies changes in the gut microbiome (Getachew *et al.* 2018). Of note, the alteration of neurotransmitter and BDNF levels is evident in depressed patients, and both play a vital role in the antidepressant actions of ketamine (Deyama and Duman 2020). Taken together, gut microbiota contributes to ketamine’s action by regulating neurotransmitters and BDNF, as shown in Fig. 6. Therefore, improving the diversity and composition of the gut microbiota could strengthen ketamine’s sustained effects.

The main advantage of knowledge graphs lies in their ability to incorporate information from disparate research literature, thereby leading to the discovery of new knowledge. In this case, a targeted investigation on ketamine and depression is a deliberate effort to gain a deeper understanding of treating mental disorders. We have demonstrated that we can retrieve meaningful results for biological questions from KetPath. Establishing a solid foundation through this research, as the knowledge graph evolves, will allow for more connections to other related aspects. This approach to constructing a pathway knowledge graph can be applied and extended to other domains, for example, by re-training models for entity recognition and relation extraction on a manually annotated database from a specific domain.

## Limitations

Our approach still leaves room for improvement. The BioKetBERT model performs well in identifying the relation between two entities in the same sentence but fails to categorize the relation as activation or inhibition because it cannot parse the relational verb between entities. One possible way to improve this approach is verb lemmatization (Rodrigues *et al.* 2017). Furthermore, developing techniques to extract pathway facts from images automatically would be a nice improvement over our manual curation efforts, for instance, by deep learning (He *et al.* 2019). This would significantly enhance scalability and open up possibilities to extract pathway knowledge from images in a vast body of literature. After retrieving knowledge from the literature and representing it in a knowledge graph, inferring new knowledge from such a graph could be improved by new techniques such as algorithmic graph analysis. For example, novel weighting schemes can be used to measure the closeness centrality and betweenness centrality of nodes on graphs, thereby identifying key entities in the pathway network.

## Conclusion

We present an approach for constructing a ketamine pathway-related knowledge graph, KetPath, from biomedical publications and public databases, including biological pathway resources for graph analysis of ketamine’s effects on brain neurotransmitters and gut microbiota. The approach combines manual fact extraction, automatic named entity recognition using CI-er, and automatic relation extraction using BioKetBERT, the latter of which we assessed to be on a par with manual relation curation. We visualized the KetPath knowledge graph at several levels to show the construction process and data architecture, demonstrating the feasibility of the approach and its biological usability. We validated the performance of KetPath by several representative query cases, highlighting that it can be used to retrieve information about biological pathways and explore potential relations between ketamine, neurotransmitters, and gut microbiota. Our approach allows users to obtain actionable results for questions at different levels of detail—from general to specific—and may be suitable for application to other drugs as well. The query cases and codes included in this work provide a helpful framework for retrieving associations from this and other knowledge graphs.

## Supplementary Material

btad771_Supplementary_Data
