# Supplementary material for: Mining literature and pathway data to explore the relations of ketamine with neurotransmitters and gut microbiota using a knowledge-graph
Source: Bioinformatics. 2023 Dec 26;40(1):btad771. doi: 10.1093/bioinformatics/btad771 (PMC10769815; doi:10.1093/bioinformatics/btad771)
Supplement: btad771_Supplementary_Data [file btad771_supplementary_data.pdf]

Bioinformatics
doi.10.1093/bioinformatics/xxxxxx

Supplementary Material

Mining literature and pathway data to explore the relations of ketamine with neurotransmitters and gut microbiota using a knowledge-graph

Ting Liu[1,2], K. Anton Feenstra[1], Jaap Heringa[1,*], Zhisheng Huang[2]

[1]Integrative Bioinformatics, Vrije Universiteit Amsterdam, 1081 HV Amsterdam, The Netherlands.
[2]Learning & Reasoning Group, Vrije Universiteit Amsterdam, 1081 HV Amsterdam, The Netherlands.

* To whom correspondence should be addressed: j.heringa@vu.nl.

S1 Bibliometric analysis in Scopus

Bibliometric data can be obtained through various search engines such as Scopus, PubMed, Web of Science, and Google Scholar (Yang and Meho, 2006), with Scopus resources being more accurate and comprehensive than other alternatives (Martín-Martín *et al.*, 2018). We therefore choose the Scopus database for bibliometric analysis and to search peer-reviewed literature with the "titles, abstracts and keywords" condition. The search inclusion and exclusion criteria were as follows: (i) the time span ranged from 2002 to 2022, encompassing 20 years in total; (ii) limit document type to article and review, whereas other document types (e.g., letters, meeting abstracts and book chapters) were excluded; (iii) the publication language was restricted to English.

The blue line in Figure S1B represents the literature related to the topic "ketamine and neurotransmitters in depression", which is obtained from the Scopus database using the code: TITLE-ABS-KEY ("ketamine" AND ("depression" OR "depressive disorder" OR "MDD") AND ("glutamate" OR "serotonin" OR "GABA" OR "dopamine" OR "histamine" OR "acetylcholine" OR "norepinephrine")) AND (LIMIT-TO (DOCTYPE, "ar") OR LIMIT-TO (DOCTYPE, "re")) AND (LIMIT-TO (LANGUAGE, "English")). The topic "gut microbiota in depression", shown in orange, is searched with the code: TITLE-ABS-KEY (("depression" OR "depressive disorder" OR "MDD") AND ("gut microbiota" OR "gut microbiome" OR "gut bacteria")) AND (LIMIT-TO (DOCTYPE, "ar") OR LIMIT-TO (DOCTYPE, "re")) AND (LIMIT-TO (LANGUAGE, "English")). The red line is associated with the topic "ketamine and gut microbiota in depression", which is retrieved with the code: TITLE-ABS-KEY ("ketamine" AND ("gut microbiota" OR "gut microbiome" OR "gut bacteria") AND ("depression" OR "depressive disorder" OR "MDD")) AND (LIMIT-TO (DOCTYPE, "re") OR LIMIT-TO (DOCTYPE, "ar")) AND (LIMIT-TO (LANGUAGE, "English")).

In the past two decades, there were 49 463 publications related to the topic "neurotransmitters in depression". Considering ketamine, the topic "ketamine and neurotransmitters in depression", 1 558 publications were retrieved, containing 908 articles and 785 reviews. This research output has shown an upward trend over the last 20 years, with the

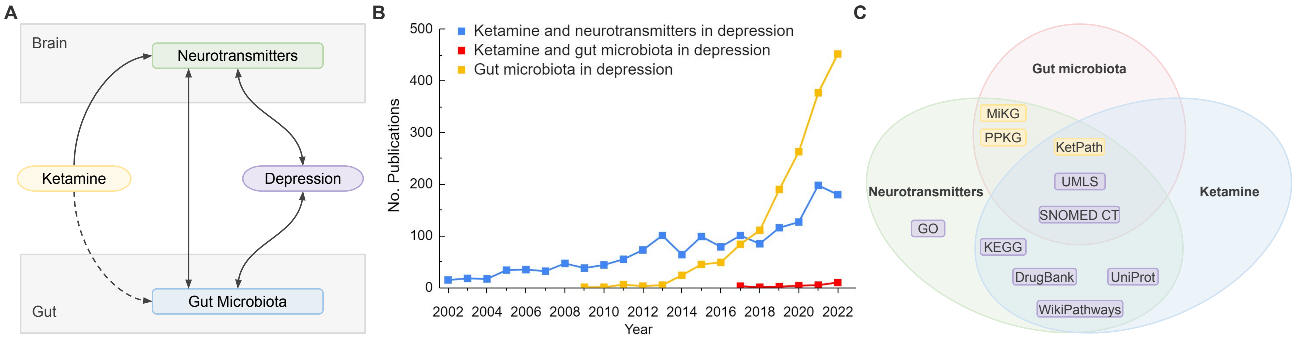

Figure S1. Research background and data sources. (A) Associations between biology concepts. (B) Research trends in related topics over the past 20 years as retrieved by Scopus (see section S1). (C) Datasets for constructing the KetPath knowledge graph. Yellow rectangles denote literature-derived datasets, while blue rectangles refer to public databases.

© The Author 2023.

number of publications increasing from 15 in 2002 to 190 in 2022, an average of 75 per year. The topic "gut microbiota in depression" started in 2009 and its research output showed an initial period (2009-2016) and a rapid development period (2017-2022). A total of 1 611 publications, including 810 articles and 801 reviews, were obtained. As shown in Figure S1B, publications related to the topic "ketamine and gut microbiota in depression" appeared in 2017 with slow development, and 25 articles were identified in a total of 8 articles and 17 reviews.

S2 Standard evaluation metrics

Recall measures how many positive cases are correctly predicted out of the total positive cases, while precision refers to the fraction of true positives from the total predicted value. F1-score is described as the harmonic mean of precision and recall. Specificity measures the proportion of true negatives. The formulas used for these four metrics are:

$$Recall = TP/(TP + FN) \tag{1}$$

$$Precision = TP/(TP + FP) \tag{2}$$

$$Specificity = TN/(TN + FP) \tag{3}$$

$$F_1 = 2 \cdot TP/(2 \cdot TP + FP + FN) \tag{4}$$

where TP refers to true positives, FP stands for false positives, TN represents true negatives, and FN means false negatives. The evaluation results of all tasks were averaged over five runs, along with their standard deviations.

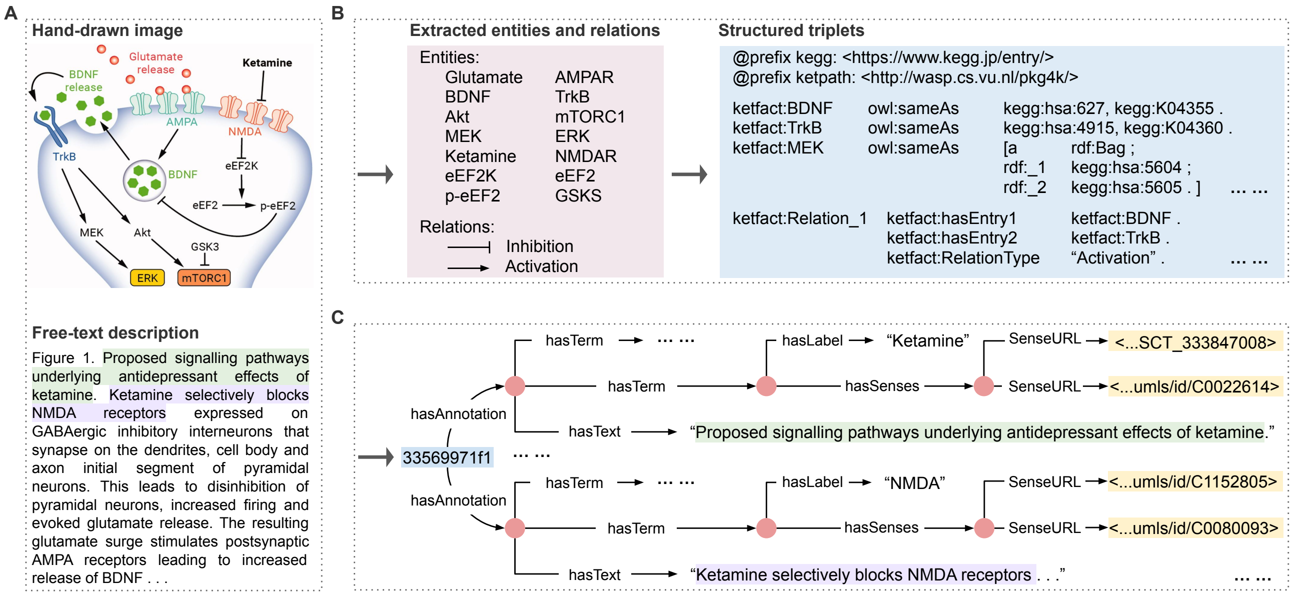

Figure S2. Pathway fact extraction and named entity recognition. (A) An example of a hand-drawn image and free text description of the ketamine pathway as described in the article with PMID 33569971 (Jelen and Stone, 2021). Highlighted sentences are returned in panel (C). (B) Manual curation of pathway facts from the image and construction of RDF triples. (C) Structural relations between the recognized named entities in sentences and their source articles, as exemplified by the two highlighted sentences in panel (A). The blue rectangle is indicating the source article and the yellow rectangles represent the URIs of the entity in UMLS and/or SNOMED CT. Red dots are customized strings.

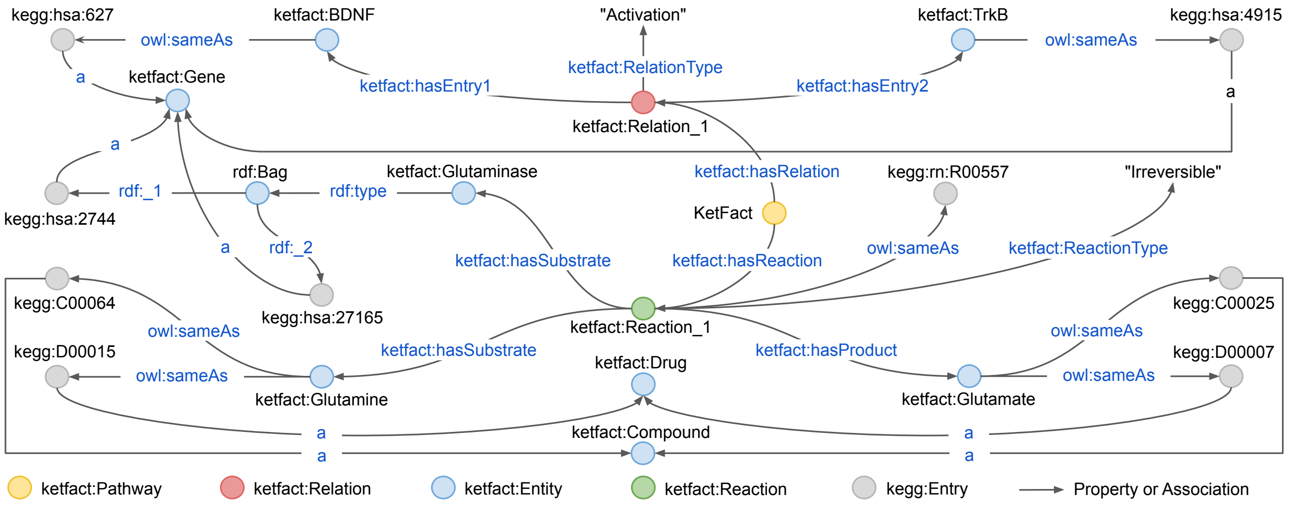

Figure S3. An example graph extracted from the KetFact knowledge base describing part of the ketamine pathway. All circle nodes are entities. Nodes with quotation marks are not entities, but free text. Arrows indicate the direction of relations from source to target.

S3 Designing questions and SPARQL queries

We retrieve information by executing semantic queries to answer natural language questions, which are designed to follow semantic logic and rules, as well as a degree of complexity. We use the SELECT form of SPARQL query language to set up semantic queries as described in (Liu *et al.*, 2022). The variables to be returned are defined using the $select$ statement, and the query conditions are specified using the $where$ statement. The $union$, $intersect$, and $except$ clauses are used to combine or exclude similar rows from multiple tables. For example, we use the $union$ clause to combine the results of two separate queries into a single set. Furthermore, the $filter$ condition limits data output based on some criteria.

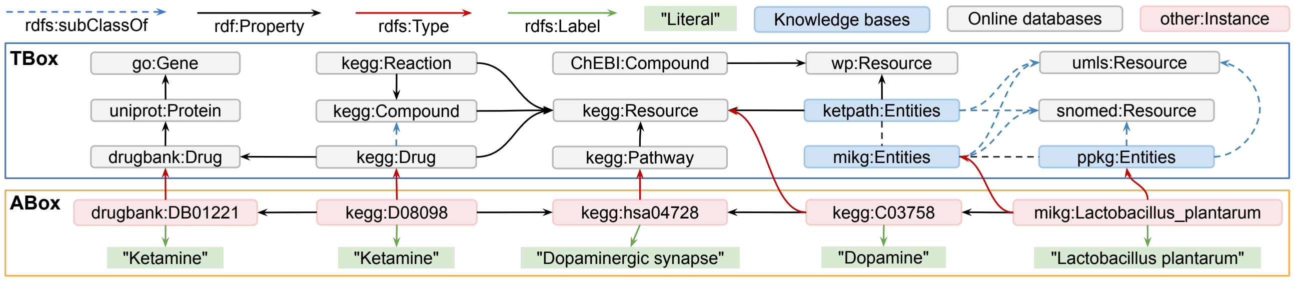

Figure S4. RDF schema showing the connections between integrated datasets. The $TBox$ consists of the terminology component of databases. The $ABox$ consists of the assertion component of databases, and different individuals are explicitly typed with corresponding classes in $TBox$. Note that all entities in the knowledge graph are mapped to existing term descriptions in other databases; however, they are only shown in text for human readability here.

Table S1. Overall statistics for datasets in the KetPath knowledge graph.

Datasets (Ref.)	Data statistics	Descriptions
KetPath (*this work*)	218,746 entities, 2 193 relations	
KetFact	36 entities, 50 relations	Manually extracted ketamine pathway facts: entities and relations
KetCept	218 710 entities, 25 280 sentences	CI-er identified named entities from pathway texts, titles and abstracts
KetRela	2 143 relations	BioKetBERT extracted relations between ketamine and neurotransmitters
MiKG (Liu *et al.*, 2020)	2 175 relations	Relations between neurotransmitters, gut microbiota and mental disorders
PPKG (Liu *et al.*, 2022)	2 307 559 entities, 446 statements	Relations between prebiotics, probiotics and human diseases
KEGG (Kanehisa *et al.*, 2014)	18 965 compounds, 70 pathways	Data profiles of pathways, genomes and chemical substances
WikiPathways (Kutmon *et al.*, 2016)	1 269 pathways	Knowledge of human biological pathways
UniProt (UniProt Consortium *et al.*, 2018)	202 771 proteins	Human protein sequences and associated detailed annotations
DrugBank (Wishart *et al.*, 2018)	11 993 drugs	Drug data with information on drug targets, pharmacology and metabolism
Gene Ontology (Gene Ontology Consortium, 2021)	5 110 terms	Gene and gene product attributes
UMLS (Bodenreider, 2004)	4 553 796 concepts	Named biomedical vocabularies and defined relationships between terms
SNOMED CT (Stearns *et al.*, 2001)	361 782 terms	Named biomedical vocabularies and defined relationships between terms

Table S2. Identified relations between ketamine and neurotransmitters by different models.

Model	Dataset	Type	Glutamate	GABA	Dopamine	Serotonin	Noradrenaline	Acetylcholine	Histamine	Total
CI-er	Whole	Sentence	824	296	391	377	208	25	22	2 143
Manual Ket	Whole	Relation	551	117	229	217	125	16	12	1 267
BioBERT	Whole	Relation	824	296	387	376	206	25	22	2 136
BioKetBERT	Whole	Relation	552	140	240	229	132	16	12	1 321
CI-er-5×CV	Test	Sentence	659±226	237±115	313±109	302±104	166±93	20±11	18±8	1 715±0
Manual Ket-5×CV	Test	Relation	441±153	94±43	183±63	174±60	100±56	13±7	10±4	1 014±41
BioBERT-5×CV	Test	Relation	609±232	203±101	259±100	263±92	143±81	14±8	14±7	1 504±166
BioKetBERT-5×CV	Test	Relation	449±134	146±76	197±80	201±72	114±66	12±7	11±6	1 130±102

Numbers of sentences and relations retrieved by query case 1. The corresponding query code is shown in Listing S1. The test set consists of 1 715 sentences, while the whole set comprises 2 143 sentences including the test set. 5×CV refers to five-fold cross-validation. Note: CI-er recognizes named entities in sentences, while BioBERT and BioKetBERT identify the relations between entities.

Table S3. **Comparison of the number of individual ketamine-neurotransmitter relations annotated by manual labelling, BioBERT, and BioKetBERT.** Relations in KetRela were derived from the articles in KetCept. Manual labelling refers to relation annotation by a group of experts. '+' and '-' indicate related and unrelated relation annotations between entities, respectively.

Dataset	Type		Glutamate	GABA	Dopamine	Serotonin	Noradrenaline	Acetylcholine	Histamine	Total
KetCept	Article		456	177	167	136	79	15	11	1 041
KetRela	Relation		824	296	391	377	208	25	22	2 143
Manual labelling	*BioBERT*	*BioKetBERT*								
+	+	+	504	113	218	206	119	14	12	1 186
+	+	−	47	4	11	10	6	2	0	80
+	−	+	0	0	0	0	0	0	0	0
+	−	−	0	0	0	1	0	0	0	1
−	+	+	48	27	22	23	13	2	0	135
−	+	−	225	152	136	137	68	7	10	735
−	−	+	0	0	0	0	0	0	0	0
−	−	−	0	0	4	0	2	0	0	6

Table S4. **Retrieving the pharmacological actions of ketamine on neurotransmitters.**

**Action**	**Target**	**Gene Name(s)**	**UniProt ID(s)**	**Reference**
Antagonist[a]	Glutamate receptor ionotropic, NMDA 3A	GRIN3A	Q8TCU5	17502428
Agonist[b]	Dopamine D2 receptor	DRD2	P14416	19391150
Inhibitor[c]	Sodium-dependent noradrenaline transporter	SLC6A2	P23975	508488
Potentiator[d]	5-HT receptor 3A	HTR3A	P46098	8777109
Antagonist	5-HT receptor 2	HTR2A,B,C	P28223, P41595, P28335	6460944
Antagonist	5-HT receptor 1	HTR1A,B,D,E,F	P08908, P28222, P28221, P28566, P30939	6460944
Binder[e]	Muscarinic acetylcholine receptor	CHRM1,2,3,4,5	P11229, P08172, P20309, P08173, P08912	8835358

Retrieved by query case 2. The corresponding query code is presented in Listing S2. Note: [a] Antagonist: Prevents the receptor from producing a response. [b] Agonist: Produces a similar response to the intended chemical and receptor. [c] Inhibitor: Blocks or distorts the active site. [d] Potentiator: Amplifies and enhances the receptor activity. [e] Binder: Inactivates the receptor site.

Table S5. **Partial results of pathway relations retrieved from the public database in the KetPath.**

Relation	R-Type	Entry 1	E1-Type	Entry 2	E2-Type
R1	Activation	cpd:C00780	Compound	hsa:3356 hsa:3357 hsa:3358	Gene
R2	Activation	hsa:3356 hsa:3357 hsa:3358	Gene	hsa:2776	Gene
R3	Activation	hsa:2776	Gene	hsa:23236 hsa:5330 hsa:5331 hsa:5332	Gene
R4	Activation	hsa:23236 hsa:5330 hsa:5331 ...	Gene	cpd:C01245	Compound
R5	Activation	cpd:C01245	Compound	hsa:3708 hsa:3709 hsa:3710	Gene
R6	Activation	cpd:C00076	Compound	hsa:7220	Gene
R7	Activation	cpd:C00780	Compound	hsa:3356 hsa:3357 hsa:3358	Gene
R8	Activation	hsa:3356 hsa:3357 hsa:3358	Gene	hsa:775 hsa:776 hsa:778 hsa:779	Gene
R9	Activation	hsa:3356 hsa:3357 hsa:3358	Gene	hsa:2776	Gene
R10	Activation	hsa:2776	Gene	hsa:23236 hsa:5330 hsa:5331 hsa:5332	Gene
R11	Activation	cpd:C01245	Compound	hsa:3708 hsa:3709 hsa:3710	Gene
R12	Activation	cpd:C00076	Compound	hsa:5578 hsa:5579 hsa:5582	Gene
R13	Activation	cpd:C00165	Compound	hsa:5578 hsa:5579 hsa:5582	Gene
R14	Activation	hsa:2776	Gene	hsa:100137049 hsa:123745 hsa:255189 hsa:5321 ...	Gene
R15	Activation	cpd:C00780	Compound	hsa:170572 hsa:200909 hsa:285242 hsa:3359 hsa:9177	Gene
R16	Activation	cpd:C00780	Compound	hsa:3360 hsa:3362 hsa:3363	Gene
R17	Activation	hsa:3360 hsa:3362 hsa:3363	Gene	hsa:2778	Gene
R18	Activation	hsa:2778	Gene	hsa:111	Gene
R19	Activation	cpd:C00575	Compound	hsa:5566 hsa:5567 hsa:5568	Gene
R20	Activation	hsa:10411	Gene	cpd:C00575	Compound

The full query results comprise 273 relations along with 471 entities, which is too large to show in detail, we present the first 20 returned results here. The directions of relations are all from Entry 1 to Entry 2.

Table S6. **List of molecular entity abbreviations in Figure 4.**

Abbreviation	Meaning	KEGG entry
Glu	Glutamate	C00025
Gln	Glutamine	C00064
D2R	Dopamine D2 receptor	hsa:1813
D3R	Dopamine D3 receptor	hsa:1814
GAD	Glutamic acid decarboxylase	ec:4.1.1.15
eEF2	Eukaryotic elongation factor 2	hsa:1938
eEF2K	eEF2 kinase	hsa:29904
BDNF	Brain derived neurotrophic factor	hsa:627
TrkB	Tropomyosin receptor kinase B	hsa:4915
mTOR	Mammalian target of rapamycin	hsa:2475
5-HT1AR	Serotonin 1A receptor	hsa:3350
VDCC	Voltage-dependent Ca^{2+} channels	hsa:773+hsa:774
ERK	Extracellular-regulated kinase	hsa:5594+hsa:5595
MAPK	Mitogen-activated protein kinase	hsa:5604+hsa:5605
ADR	Adrenergic receptor	hsa:146+hsa:147+hsa:148
Akt	Protein kinase B	hsa:10000+hsa:207+hsa:208
vGlut	Vesicular glutamate transporter	hsa:246213+hsa:57030+hsa:57084
AMPAR	α-amino-3-hydroxy-5-methyl-4-isoxazolepropionic acid receptor	hsa:2890+hsa:2891+hsa:2892+hsa:2893
NMDAR	N-methyl-D-aspartate receptor	hsa:116443+hsa:116444+hsa:2902+hsa:2903+hsa:2904+hsa:2905+hsa:2906
$GABA_AR$	GABA type A receptor	hsa:2554+hsa:2555+hsa:2556+hsa:2557+hsa:2558+hsa:2559+hsa:2560+hsa:2561+ hsa:2562+hsa:2563+hsa:2564+hsa:2565+hsa:2566+hsa:2567+hsa:2568+hsa:55879

Listing S1. Query protocol for query case 1 to retrieve relations between ketamine and neurotransmitters.

```
prefix rdf: <http://www.w3.org/1999/02/22-rdf-syntax-ns#>
prefix owl: <http://www.w3.org/2002/07/owl#>
prefix snomed: <http://wasp.cs.vu.nl/sct/sct#>
prefix rdfs: <http://www.w3.org/2000/01/rdf-schema#>
prefix ketcept: <http://wasp.cs.vu.nl/ketcept/>
prefix ketrela: <http://wasp.cs.vu.nl/ketrela/>

select distinct  ?text  ?termLabel1  ?termLabel2  ?Relation  ?Text2
      ?Entity1  ?Entity2  ?manualLabel  ?BioKetBERTLabel

where {
{?PMID          ketcept:hasAnnotation     ?Annotation .
?Annotation     ketcept:hasText           ?Text .
?Annotation     ketcept:hasTerm           ?Term1 .
?Term1          ketcept:hasLabel          ?TermLabel1 .
?Term1          ketcept:hasSenses         ?Senses1 .
?Senses1        ketcept:SenseURL          ?URL .
?URL            snomed:hasEnglishLabel    ?URLLabel .
?URL            rdfs:subClassOf    <http://www.ihtsdo.org/SCT_35069000> .}
filter exists
{?Annotation    ketcept:hasTerm           ?Term2 .
?Term2          ketcept:hasLabel          ?TermLabel2 .
?Term2          ketcept:hasSenses         ?Senses2 .
?Senses2        ketcept:SenseURL   <http://www.ihtsdo.org/SCT_373464007> .}
union
{?Relation      ketrela:hasText           ?Text2 ;
                ketrela:hasEntity1        ?Entity1 ;
                ketrela:hasEntity2        ?Entity2 ;
                ketrela:manualLabel       ?manualLabel ;
                ketrela:BioBERTLabel      ?BioBERTLabel ;
                ketrela:BioKetBERTLabel   ?BioKetBERTLabel .}
 }
```

Listing S2. Query protocol for query case 2 to retrieve ketamine's actions and neurotransmitter pathways from public databases.

```
prefix rdf: <http://www.w3.org/1999/02/22-rdf-syntax-ns#>
prefix owl: <http://www.w3.org/2002/07/owl#>
prefix snomed: <http://wasp.cs.vu.nl/sct/sct#>
prefix rdfs: <http://www.w3.org/2000/01/rdf-schema#>
prefix db: <http://www.drugbank.ca/>
prefix dcterms: <http://purl.org/dc/terms/>
prefix wp: <http://vocabularies.wikipathways.org/wp#>
prefix kegg: <https://www.kegg.jp/dbget-bin/www_bget?>

select distinct ?Action  ?TargetName  ?GeneName  ?PeptideID  ?PMID
      ?PathwayName  ?PathwayTitle  ?PathwayLink  ?Relation  ?R-Type
      ?E1Name  ?E1Type  ?E1Definition  ?E2Name  ?E2Type  ?E2Definition

where {
{?DBID          db:Name                   `Ketamine' ;
                db:externalIdentifiers    ?EIDs ;
                db:targets                ?Targets .
?EIDs           db:resource               `KEGG ;
                db:resourceID             ?EID .
?Targets        db:target                 ?Target .
?Target         db:targetID               ?TargetID ;
                db:targetName             ?TargetName ;
                db:actions                ?Actions ;
                db:references             ?References ;
                db:polypeptide            ?Polypeptide .
?Actions        db:action                 ?Action .
?References     db:articles               ?Article .
?Article        db:pubmedID               ?PMID .
?Polypeptide    db:peptideID              ?PeptideID ;
                db:peptideSource          ?PeptideSource ;
                db:geneName               ?GeneName .}
union
{?Pathway       kegg:pathwayName          ?PathwayName ;
                kegg:pathwayTitle         ?PathwayTitle ;
                kegg:pathwayLink          ?PathwayLink ;
                kegg:Entry                ?Entry ;
                kegg:Relation             ?Relation.
?Relation       kegg:hasEntry1            ?Entry1 ;
                kegg:hasEntry2            ?Entry2 ;
                kegg:hasRelationSubtype   ?R-Type .
?Entry          kegg:entryID              ?Entry1 ;
                kegg:entryName            ?E1Name ;
                kegg:entryType            ?E1Type ;
                kegg:entryLink            ?E1Link .
?E1Link         kegg:hasDefinition        ?E1Definition .
?Entry          kegg:entryID              ?Entry2 ;
                kegg:entryName            ?E2Name ;
                kegg:entryType            ?E2Type ;
                kegg:entryLink            ?E2Link .
?E2Link         kegg:hasDefinition        ?E2Definition .
filter regex    (?PathwayTitle,`synapse',`i')}
union
{?geneProduct   a                         wp:GeneProduct .
?geneProduct    rdfs:label                ?label .
?geneProduct    dcterms:isPartOf          ?pathway .
?pathway        rdf:type                  ?type .
?pathway        wp:source                 ?source .
?source         rdfs:label                ?sourceLabel.
?pathway        wp:target                 ?target .
?target         rdfs:label                ?targetLabel .
?pathway        dcterms:isPartOf          ?partpath .
?partpath       dc:title                  ?title .
?partpath       dc:identifier             ?identifier .
?partpath       wp:organismName           `Homo sapiens' .
filter regex    (str(?sourceLabel), `SLC6A2').}
}
```

Listing S3. Query protocol for query case 3 to retrieve pathway relations for ketamine in the literature.

```
prefix rdf: <http://www.w3.org/1999/02/22-rdf-syntax-ns#>
prefix owl: <http://www.w3.org/2002/07/owl#>
prefix snomed: <http://wasp.cs.vu.nl/sct/sct#>
prefix rdfs: <http://www.w3.org/2000/01/rdf-schema#>
prefix ketfact: <http://wasp.cs.vu.nl/ketfact/>

select distinct ?Type ?Entry1 ?E1Link ?Entry2 ?E2Link ?Item1
    ?I1Link ?Item2 ?I2Link

where {
{?Relation   ketfact:RelationType   ?Type ;
             ketfact:hasEntry1      ?Entry1 ;
             ketfact:hasEntry2      ?Entry2 ;
?Entry1      ketfact:hasName        ?E1Name ;
             owl:sameAs             ?E1Link .
?Entry2      ketfact:hasName        ?E2Name ;
             owl:sameAs             ?E2Link .}
union
{?Relation   ketfact:RelationType   ?Type ;
             ketfact:hasEntry1      ?Entry1 ;
             ketfact:hasEntry2      ?Entry2 ;
?Entry1      ketfact:hasName        ?E1Name ;
             owl:sameAs             ?E1Link .
?Item2       rdfs:subClassOf        ?Entry2 ;
             ketfact:hasName        ?I2Name ;
             owl:sameAs             ?I2Link .}
union
{?Relation   ketfact:RelationType   ?Type ;
             ketfact:hasEntry1      ?Entry1 ;
             ketfact:hasEntry2      ?Entry2 ;
?Item1       rdfs:subClassOf        ?Entry1 ;
             ketfact:hasName        ?I1Name ;
             owl:sameAs             ?I1Link .}
?Entry2      ketfact:hasName        ?E2Name ;
             owl:sameAs             ?E2Link .}
union
{?Relation   ketfact:RelationType   ?Type ;
             ketfact:hasEntry1      ?Entry1 ;
             ketfact:hasEntry2      ?Entry2 .
?Item1       rdfs:subClassOf        ?Entry1 ;
             ketfact:hasName        ?I1Name ;
             owl:sameAs             ?I1Link .}
?Item2       rdfs:subClassOf        ?Entry2 ;
             ketfact:hasName        ?I2Name ;
             owl:sameAs             ?I2Link .}
}
```

Listing S4. Query protocol for query case 4 to retrieve relations between ketamine and gut microbes.

```
prefix rdf: <http://www.w3.org/1999/02/22-rdf-syntax-ns#>
prefix owl: <http://www.w3.org/2002/07/owl#>
prefix snomed: <http://wasp.cs.vu.nl/sct/sct#>
prefix rdfs: <http://www.w3.org/2000/01/rdf-schema#>
prefix mikg: <http://wasp.cs.vu.nl/mikg/>
prefix ketpath: <http://wasp.cs.vu.nl/ketpath/>
prefix ppconcept: <http://wasp.cs.vu.nl/ppconcept#>
prefix ppstatement: <http://wasp.cs.vu.nl/ppstatement#>

select distinct ?Statement ?pmid ?doi ?Neurotransmitter
    ?Microbiota ?probiotics ?composition ?text ?URLLabel

where {
{?statement   mikg:hasNeurotransmitter     ?Neurotransmitter ;
              mikg:hasGutMicrobiota        ?Microbiota ;
              mikg:hasModulation           ?Modulation ;
              mikg:hasReference            ?Reference .
?Reference    mikg:hasPMID                 ?pmid ;
              mikg:hasDOI                  ?doi .}
union
{?statement   ppstatement:hasEffectOn      ?Effect ;
              ppstatement:hasProbiotics    ?Probiotics ;
              ppstatement:hasReference     ?Reference .
?reference    ppstatement:hasPMID          ?pmid .
filter regex  (?effect,`bdnf',`i')}
union
{?Statement   ppstatement:hasEffectOn      ?Effect ;
              ppstatement:hasProbiotics    ?Probiotics ;
              ppstatement:hasReference     ?Reference ;
?Reference    ppstatement:hasPMID          ?pmid .
?Probiotics   a                            owl:Class ;
              owl:intersectionOf           ?Mixture .
?Mixture      rdf:rest*/rdf:first          ?Composition .
filter regex  (?effect,`bdnf',`i')}
union
{?pmid        ppconcept:hasAnnotations     ?Annotations .
?Annotations  ppconcept:hasText            ?Text .
?Annotations  ppconcept:hasAnnotation      ?Annotation .
?Annotation   ppconcept:hasTerm            ?Term .
?Term         ppconcept:hasSenses          ?Senses .
?Senses       ppconcept:hasSense           ?Sense .
?Sense        ppconcept:SenseURL           ?URL .
              rdfs:subClassOf   <http://www.ihtsdo.org/SCT_264395009> .
?URL          snomed:hasEnglishLabel       ?URLLabel .
filter regex  (?Text,`bdnf',`i')}
}
```

References

Bodenreider, O. (2004). The unified medical language system (UMLS): integrating biomedical terminology. *Nucleic acids research*, **32**(suppl_1), D267–D270.

Gene Ontology Consortium (2021). The Gene Ontology resource: enriching a gold mine. *Nucleic Acids Research*, **49**(D1), D325–D334.

Jelen, L. A. and Stone, J. M. (2021). Ketamine for depression. *International Review of Psychiatry*, **33**(3), 207–228.

Kanehisa, M. *et al.* (2014). Data, information, knowledge and principle: back to metabolism in kegg. *Nucleic acids research*, **42**(D1), D199–D205.

Kutmon, M. *et al.* (2016). Wikipathways: capturing the full diversity of pathway knowledge. *Nucleic acids research*, **44**(D1), D488–D494.

Liu, T. *et al.* (2020). Exploring the microbiota-gut-brain axis for mental disorders with knowledge graphs. *Journal of Artificial Intelligence for Medical Sciences*.

Liu, T. *et al.* (2022). Towards a knowledge graph for pre-/probiotics and microbiota–gut–brain axis diseases. *Scientific Reports*, **12**(1), 1–11.

Martín-Martín, A. *et al.* (2018). Google scholar, web of science, and scopus: A systematic comparison of citations in 252 subject categories. *Journal of informetrics*, **12**(4), 1160–1177.

Stearns, M. Q. *et al.* (2001). SNOMED clinical terms: overview of the development process and project status. In *Proceedings of the AMIA Symposium*, p 662. American Medical Informatics Association.

UniProt Consortium *et al.* (2018). Uniprot: the universal protein knowledgebase. *Nucleic acids research*, **46**(5), 2699.

Wishart, D. S. *et al.* (2018). DrugBank 5.0: a major update to the DrugBank database for 2018. *Nucleic acids research*, **46**(D1), D1074–D1082.

Yang, K. and Meho, L. I. (2006). Citation analysis: a comparison of google scholar, scopus, and web of science. *Proceedings of the American Society for information science and technology*, **43**(1), 1–15.
